# The Effect of Dentifrice on Micro-Hardness, Surface Gloss, and Micro-Roughness of Nano Filled Conventional and Bulk-Fill Polymer Composite—A Micro Indentation and Profilometric Study

**DOI:** 10.3390/ma15124347

**Published:** 2022-06-20

**Authors:** Mashael Binhasan, Abdulilah H. Solimanie, Khalid S. Almuammar, Ahmed R. Alnajres, Mai M. Alhamdan, Khold Al Ahdal, Yasser F. Alfaawaz, Khalid Ali, Fahim Vohra, Tariq Abduljabbar

**Affiliations:** 1 Department of Restorative Dental Sciences College of Dentistry, King Saud University, Riyadh 11421, Saudi Arabia; mbinhasan@ksu.edu.sa (M.B.); kalahdal@ksu.edu.sa (K.A.A.); yalfaawaz@ksu.edu.sa (Y.F.A.); 2 Department of General Dentistry, College of Dentistry, King Saud University, Riyadh 11421, Saudi Arabia; abdalulah555@hotmail.com (A.H.S.); khalid.muammar@gmail.com (K.S.A.); a.alnjers@gmail.com (A.R.A.); 3 Department of Prosthetic Dental Science, College of Dentistry, King Saud University, Riyadh 11421, Saudi Arabia; mayalhamdan@ksu.edu.sa (M.M.A.); fvohra@ksu.edu.sa (F.V.); 4 Department of Clinical Sciences, College of Dentistry, King Saud University, Riyadh 11421, Saudi Arabia; khalid.alires@gmail.com

**Keywords:** dentifrice, polymer composite, bulk-fill composite, nanostructure, micro-hardness, gloss, surface roughness

## Abstract

The aim of the present study was to evaluate the effect of brushing with two whitening dentifrices (Colgate Optic White (COW) and Colgate Optic White Charcoal (COC)) on surface gloss, micro-roughness, and micro-hardness of nanostructured hybrid conventional (Z350) and bulk-fill (Tetric N Ceram bulk-fill) polymer composite. In total, 96 disk samples using two nano-hybrid composite polymers (Z350 and Tetric N Ceram Bulk-fill) were prepared. All specimens were exposed to two different dentifrices (COW and COC), resulting in four main subgroups in the study. Specimens were assessed for surface gloss, micro-roughness (Ra), and micro-hardness using standardized methodology. Means and standard deviations of properties compared using paired t-test, one-way and two-way ANOVA, and post hoc test. The presence of dentifrices did not show any significant difference in micro-hardness values of Z350 (*p* > 0.05), whereas micro-hardness of bulk-fill composite significantly reduced on dentifrices exposure (*p* ≤ 0.05). Bulk-fill polymer composite showed significant reduction in gloss after dentifrice exposure (*p* < 0.05), however, Z350 showed no significant loss of gloss due to dentifrices (*p* > 0.05). A significant increase in Ra was observed for both resin materials after exposure to dentifrices (COC and COW). Conventional resin composite (Z350) showed comparable surface hardness and gloss before and after dentifrice exposure, however, micro-roughness increased significantly due to dentifrice exposure. Bulk-fill resin (Tetric N Ceram) showed significant loss of micro-hardness and gloss and increase in micro-roughness on dentifrice exposure. Conventional nano-hybrid composite polymer showed better durability in resisting loss of surface properties compared to bulk-fill resin polymer in the present experiment.

## 1. Introduction

The American Dental Association (ADA) has approved an array of dentifrices for oral prophylaxis, however, an ideal dentifrice should remove plaque and stains efficiently without causing tooth abrasion [1,2]. A multitude of whitening dentifrices can be purchased from pharmacies, to provide fast, easy, and low-cost whitening effect. The mechanisms in which these dentifrices whiten the teeth varies depending on the dentifrice constituents present [3]. Historically, the main component in whitening toothpastes are abrasive substances, including calcium carbonate and silica as over-the-counter dentifrices [4]. As dentifrices are inherently abrasive to tooth and restorations, they cause an increase in surface roughness and a decrease in surface gloss of the tooth and restoration. This can further increase susceptibility to plaque accumulation and compromised aesthetics [5].

Resin based nano-hybrid composites are considered as materials of choice due to their mechanical and aesthetic properties in dentistry [6]. Recent advances with improved material mechanical, biological, and physical properties have supported the widespread use of nano-hybrid composites [7]. Nanostructured polymer composites are available in variety of matrix formulations along with different filler types (silica, alumina, titanium dioxide, and zirconia), which influences their handling and physical properties [8,9,10]. In recent times, bulk-fill nano-composites have gained marked recognition and application due to cost effectiveness and dental-operator convenience. Bulk-fill resin composites allow for composite placement in high thickness (4 mm or above) to avoid incremental placement, without compromising the polymerization shrinkage, cavity adaptation, or degree of conversion [11].

A satisfactory clinical performance and longevity of any tooth-color restoration is highly influenced by its ability to resist surface degradation in the oral environment [12]. Among various properties, surface quality, i.e., surface roughness, gloss, and surface microhardness, are considered critical in the successful prognosis of resin restorations in the clinical setting [13]. Surface roughness and gloss also influence the optical properties of composite restoration. Studies evaluating the effect of whitening dentifrices on surface roughness of composite resins have shown varying outcomes, due to different abrasives [14,15].

According to available indexed literature, data related to the effect of different whitening dentifrice on the surface properties, i.e., gloss retention, surface roughness, and microhardness, of a nano-hybrid conventional and bulk-fill polymer composite is limited. Therefore, it was postulated that there would be no significant difference on tested nano filled composites (Z350 and Tetric N Ceram bulk-fill) gloss, surface roughness, and microhardness after exposure to different dentifrice (Colgate Optic White (COW) and Colgate Optic Charcoal (COC)) used with tooth-brushing. Therefore, the aim of the present study was to evaluate the effect of brushing with two whitening dentifrices (COW and COC) on surface gloss, micro roughness, and microhardness of nano filled conventional and bulk-fill polymer based composites.

## 2. Materials and Methods

In total, 96 disk samples using two different nanostructured composite resins, i.e., conventional composite Z350 (Filtek Z350 XT/3M ESPE, St. Paul, MN, USA) and a light-cured bulk-fill nano filled composite (Tetric N Ceram Bulk-fill/Ivoclar Vivadent AG, Schaan, Liechtenstein) were prepared. In total, 48 disks had 2 mm height and 10 mm diameter and were formed with conventional composite (Filtek Z350 XT/3M ESPE, St. Paul, MN, USA). A further 48 prepared disks had 4mm height and 10 mm diameter, formed from bulk-fill composite materials (Tetric N Ceram Bulk-fill/Ivoclar Vivadent AG, Schaan, Liechtenstein) using a circular mold. These two composite material groups were further divided in to three separate sub-groups with 16 specimens in each for later testing of properties (Gloss, micro-roughness, and micro-hardness). In each sub-group, specimens were divided into two halves (*n* = 8) on the basis of exposure to two different dentifrice (Table 1). The two dentifrices included were Colgate Optic White (COW) and Colgate Optic White Charcoal (COC) (Colgate-Palmolive, New York, NY, USA) (Table 1). Surface properties, i.e., Gloss, micro-roughness, and micro-hardness were assessed on the four groups mentioned below

Group 1 (ZW): Z350 and Colgate Optic White (COW)

Group 2 (ZC): Z350 and Colgate Optic Charcoal (COC)

Group 3 (TW): Tetric N Ceram bulk-fill and COW

Group 4 (TC): Tetric N Ceram bulk-fill and COC

### 2.1. Specimens Preparation

A circular mold was placed on a transparent matrix strip supported by a glass slide and overfilled with composite resin. The top of the mold was covered by another matrix strip and glass slide. Both the top and bottom surfaces of the disks were polymerized through the glass slides with a high-intensity source of blue light (Eliphar S10, 3M ESPE, St. Paul, MN, USA). According to the manufacturer, all the specimens were polymerized from both top and bottom for 20 s separately. Specimens were removed from their molds and immersed in distilled water at 37 °C for 24 h in dark containers before any measurements was performed. After 24 h of storage, the initial gloss, micro-roughness, and microhardness measurements were performed.

### 2.2. Brushing Protocol

Composite disks were brushed using Ultradent brushing (Ultradent products, South Jordan, UT, USA) unit at a speed of 2.5 cm/s for 25,000 reciprocal strokes, 60 strokes per minute. Considering an average of twice daily brushing, and total 5 s brushing exposure daily to the restoration [16], the study included 13.5 years of brushing exposure. While the sample holder rotated, the toothbrushes moved horizontally, back and forth. Soft toothbrushes (Colgate 3608 Soft, Colgate Oral Pharmaceuticals Inc., New York, NY, USA) with 2 different dentifrice (Colgate Optic White, and Colgate Toothpaste Optic White Charcoal; Colgate-Palmolive company, New York, NY, USA) solutions of 50 g of toothpaste to 80 mL of deionized water was used to brush the composite disks with a 180 g force.

### 2.3. Micro-Hardness Assessment

In total, 16 disks from each polymer composite material were further subdivided into 2 groups on the basis of dentifrice (*n* = 8). Among the four study groups, all disks were exposed to two study dentifrices in combination with tooth-brushing cycles. The specific surface of all specimens was subjected to Vickers micro-indentations (five indents), before and after dentifrice exposure and simulated brushing. The microhardness was measured with a micro indentation tester (INNOVATEST, Maastricht, Netherlands) with a pyramidal diamond shaped Vickers indenter tip, at 5 N load for 20 s [17]. The means of Vickers Hardness Number (VHN) for each specimen was identified.

### 2.4. Gloss Retention

A total of 32 disks, 16 disks from each composite materials, were further subdivided into 2 groups on the basis of dentifrice (*n* = 8). The surface gloss was measured using Novo-Curve gloss meter (Rhopoint instruments Ltd., Hastings, Sussex, UK) on the top surface of each specimen (five measurements per specimen). The device has a 5 mm aperture and was calibrated (0.5 GU) with a plate and was assigned a value of 100.4, as per manufacturer’s instructions, prior to measurements. All the specimens in four groups were exposed to a different brushing regime (assigned) and positioned over the aperture of the gloss meter. The gloss measurements were performed before and after exposure to dentifrice.

### 2.5. Surface Roughness Assessment

Eight specimens were allocated in each subgroup (*n* = 8) (Groups 1 to 4) and surface roughness (Ra) was recorded at 3 different points selected on each specimen. The Ra was measured using a profilometer (model SJ-401, Mitutoyo, Kawasaki, Japan), which was calibrated prior to the assessment. A total of 3 measurements were obtained for each specimen and the arithmetic mean was calculated. This procedure was performed before and after simulated brushing with two dentifrices included in the study.

### 2.6. Statistical Analysis

Normality of data was assessed using Kolmogorov–Smirnov and Shapiro–Wilk tests. The obtained data was analyzed using Statistical Program for Social Sciences (SPSS, Version 22, IBM, New York, NY, USA). Means and standard deviations of micro-hardness, gloss, and micro-roughness were compared between pre- and post-exposure were compared using paired *t*-test and repeated measure two-way ANOVA. The overall effect of material and dentifrice were compared using two-way ANOVA. Repeated measure ANOVA and post hoc multiple comparisons test were also applied for comparing the individual study groups for microhardness, gloss, and micro-roughness.

## 3. Results

The data obtained for microhardness, gloss, and surface roughness showed normal distribution.

### 3.1. Microhardness (VHN)

**Figure 1:**
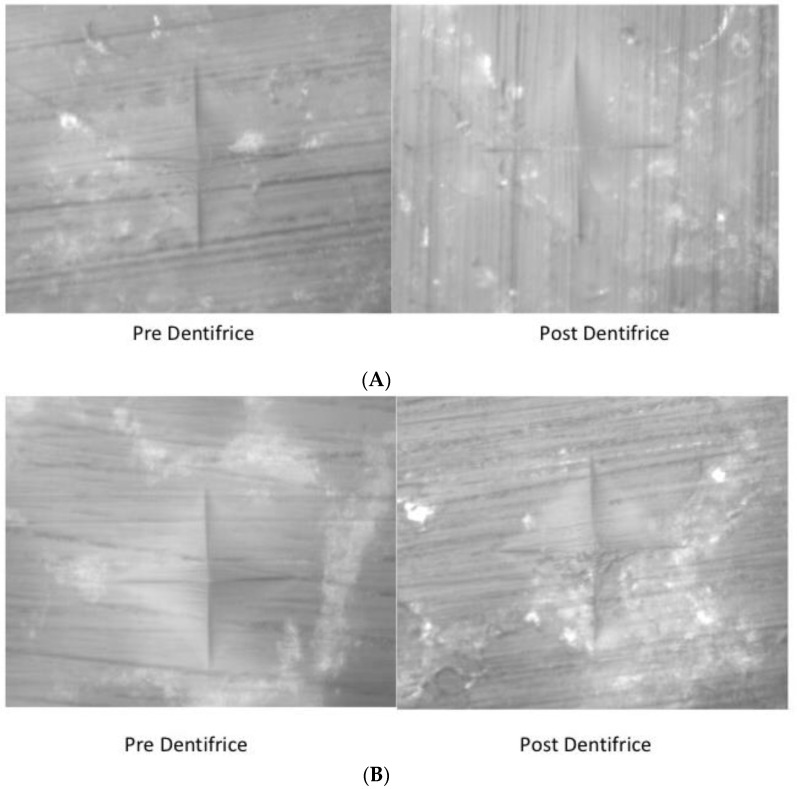
Vickers indentations for hardness testing before and after dentifrice exposure in conventional resin (**A**) and bulk-fill resin (**B**).

For Z350, ZW specimens before exposure to COW displayed the highest mean value for microhardness (75.54 ± 7.46). Among Z-350 samples, ZC after getting exposed to COC group displayed the minimum mean values (69.5 ± 6.13). Exposure to different dentifrices did not show any significant different in microhardness values of Z350 composite. Mean difference of Z350 composite sample before and after exposure to COW and COC was found to be 5.35 and 3.45, respectively (Table 2) (Figure 1).

For Tetric N Ceram, the highest mean value was demonstrated by TW specimens before exposure to dentifrice (59.19 ± 5.58). The minimum value of microhardness was for samples exposed to COW and COC dentifrices (46.03 ± 3.16 and 46.02 ± 3.15, respectively). Microhardness significantly reduced after exposure to dentifrices compared to baseline (*p* ≤ 0.05). No significant difference was observed among the four groups for post-exposure microhardness (*p* ≥ 0.05) (Table 3). The loss of microhardness was significantly different among the study groups (*p* ≤ 0.05) (Table 3). Specimens in group 1 and group 2 displayed significantly higher microhardness as compared to group 3 and group 4 (Table 4).

Employing two-way ANOVA, it was observed that micro-hardness (VHN) was significantly influenced by the change in materials (*p* < 0.000). Micro hardness among Tetric N Ceram samples, significantly reduced due to exposure to dentifrice, however, a change in dentifrice type overall (comparing all four groups) did not have any significant influence on micro-hardness (VHN). In addition, there was no interaction between the two factors (material and dentifrice) (Table 5).

### 3.2. Surface Gloss

In Z350 material, group 2 specimens before COC exposure displayed the highest mean value of gloss (163.06 ± 6.85). However, group 1 specimens after exposure to COW displayed the minimum mean values (154.31 ± 9.66). It was also found that there was no significant difference in gloss for Z350 after exposure to COC and COW (*p* ≥ 0.05). The mean difference in gloss following COW and COC exposure was 4.75 and 8.13, respectively. For Tetric N Ceram composite, group 4 pre-exposure to COC had the highest gloss (163.58 ± 7.27). The least gloss was presented by group 3 following COW treatment (143.15 ± 8.77). Mean gloss for group 3 and group 4 exposed to COW and COC were comparable (*p* ≤ 0.05). Mean gloss difference for TW and TC specimens was 17.7 and 12.89, respectively (Table 6). Mean gloss among the study groups after dentifrice exposure was significantly different (*p* = 0.041) (Table 6). Mean gloss in group 3 was significantly lower than specimens of groups 1, 2, and 4, respectively (*p* < 0.05) (Table 7). Mean gloss among groups 1, 2, and 4 were comparable (Table 8).

Employing two-way ANOVA, it was observed that gloss was significantly influenced by the change in materials (*p* < 0.000), however, a change in dentifrice overall did not have any significant influence on micro-hardness (VHN). In addition, there was no interaction between the two factors (material and dentifrice) (Table 9). A significant reduction in surface gloss (*p* < 0.05) (Table 6) due to both dentifrices was only observed in Tetric N Ceram material. The reduction in gloss for Z350 due to dentifrice exposure was statistically comparable (*p* > 0.05) (Table 6).

### 3.3. Surface Roughness

**Figure 2:**
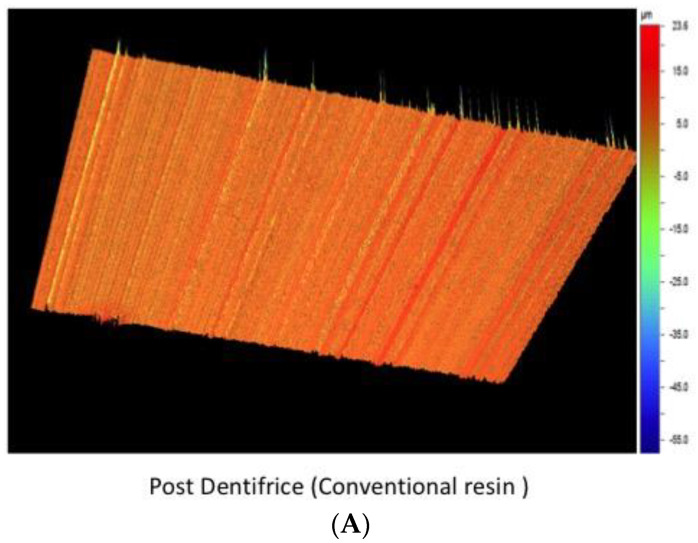

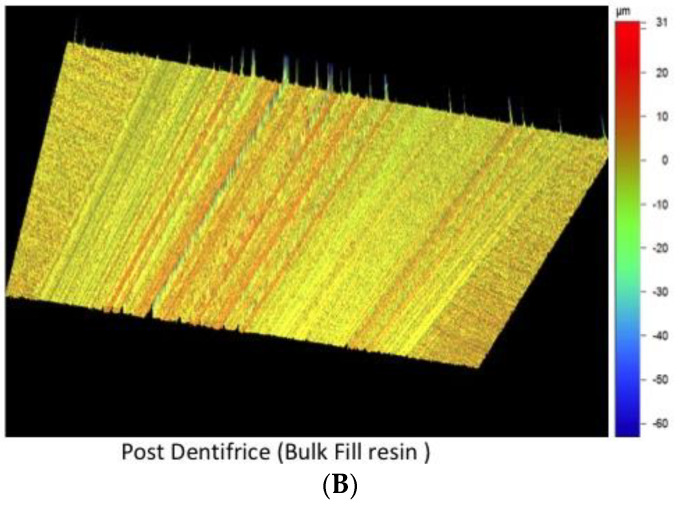
Surface roughness (Ra) micrograph after dentifrice exposure in conventional composite (**A**) and bulk-fill resin (**B**).

For Z350, group 2 exposed to COC displayed the highest mean Ra value (1.01 ± 0.33). However, group 2 specimens displayed the minimum mean Ra values (0.68 ± 0.29). Exposure to dentifrice showed increase in Ra for of Z350 samples (*p* ≤ 0.05) (Table 8) (Figure 2). For Tetric N ceram material, a significant increase in Ra was observed after dentifrice exposure (*p* ≤ 0.05). The mean difference for TW and TC in Ra was 0.32 and 0.21, respectively (Table 10). A significant difference was observed among Ra values of the study groups (ZW, ZC, TW, and TC) (*p* ≤ 0.05) (Table 11). Group 2 and group 4 specimens showed significantly higher mean Ra than group 1 (*p* < 0.05) (Table 12). However, group 4 showed comparable Ra to specimens in groups 3 and 2 specimens, respectively (*p* > 0.05).

It was observed that although surface roughness increased for both materials (Z350 and Tetric N Ceram) after exposure to both dentifrices (COW and COC) (Table 10), the overall effect of material and dentifrice types (two-way ANOVA) did not show any significant influence on the surface roughness (*p* > 0.05). In addition, there was no interaction between the two factors (material and dentifrice) (Table 13).

## 4. Discussion

The present study was based on the hypothesis that that there will be no significant difference in gloss, surface roughness, and microhardness in Z350 and Tetric N Ceram bulk-fill after exposure to different dentifrice (Colgate Optic White and Colgate Optic Charcoal) through tooth-brushing. From the results of the existing study it was found that the postulated hypothesis was rejected as gloss, surface roughness, and microhardness values were altered after exposure to dentifrice via tooth-brushing.

In the present study a motorized toothbrush was used in order to standardize the uniform brushing pattern of strokes. Literature has revealed that toothbrush hardness can alter the surface properties of tooth-colored restorations [17,18]. Therefore, to avoid any negative impact, a soft bristle toothbrush was used. However, a toothbrush simulator would deliver better uniform strokes along with constant pressure [19]. Moreover, in order to limit any variation, brushing of all the disks was performed by a single operator. In addition, the prepared samples were immersed and stored in water rather than artificial saliva, as it forms surface protective layer on the composite disks and may affect the surface properties [20]. The VHN indentation test was used to measure the microhardness of the restorative material, as its calculations are independent of the size of the indenter. In addition, it is indicated and commonly used for macro and micro hardness testing for dental materials [16].

Composite resins are more prone to chemical alteration compared with inert metal or ceramic restorations due to their organic matrix [21]. Surface hardness of composite restoration is associated with the mechanical performance of dental restorations. Surface characteristics are dependent on the degree of conversion of monomer to polymer, size, weight, and volume of the fillers, and composition of the organic matrix [22]. In the present study, bulk-fill nano filled composite demonstrated that surface microhardness decreased after tooth-brushing with either of the dentifrices. Moreover, it was also found that nano-hybrid Tetric N Ceram bulk-fill composite disks displayed significant difference in the surface micro-hardness after exposure to both the dentifrices as compared to their Z350 counterpart. Previous studies have identified the correlation between increase in surface roughness of polymer-based composites after tooth-brushing with dentifrice [23,24]. These findings can be explained by the difference in filler size and monomer composition of different composites. This may also be due to low initial microhardness value of bulk-fill composite, which accounts for the significant difference in Tetric N Ceram bulk-fill composites [24]. Moreover, the Z350 composite which consists of zirconia and silica particles in nanocluster form, results in decreasing interstitial spaces between the nanoparticles, which explains why this composite exhibited significant difference in surface roughness value before and after exposure to dentifrice [22]. In addition, in the author’s opinion these findings also suggest that difference in abrasiveness of the dentifrice does not affect the outcomes of the existing study. It is the composition of the polymeric composite nano filled material which is associated with the change in surface roughness after tooth-brushing abrasion with the dentifrice. In addition, the presence of hydrogen peroxide (COW) and sodium lauryl sulphate (COC) (Table 1) can be associated with the surface changes (surface roughness) of resin composites.

Regarding surface gloss, results of the present study revealed that gloss among the nano filled material (Tetric N Ceram) decreased after tooth-brushing using dentifrice. Literature claims that the surface gloss of the composite restoration showed an inverse relation with surface roughness [25,26]. This finding can be attributed to change acquired by the surface of bulk-fill nano filled polymer after dentifrice use resulting in decreased incidence and reflection of light, which decreases the surface gloss. The difference in gloss value after dentifrice use was only observed with Tetric N Ceram bulk-fill nano filled polymer. This is attributed to the predominant barium glass, flourosilicates, and Ytterbium Triflourides as filler content of the material (Table 1). These results are in line with the study by O’Neill et al. and Shimokawa et al. [13,27]. In addition, this can also be explained by the particle size and asymmetrical shape of the filler particles of Tetric N Ceram, resulting in decreased homogeneity of matrix and filler complex, thus contributing to less surface gloss [26]. These findings also suggest that the decrease in surface gloss was associated with the type of composite rather than the dentifrice used.

Regarding surface roughness, in the present study both the composites when exposed to two different dentifrices displayed significant increase in surface roughness as compared to the disks not exposed to dentifrice. Surface roughness (Ra) is attributed to the larger and protruded filler particles present in the matrix, exposed due to displacement of surrounding filler by toothbrush abrasion [28,29]. This causes micro crack generation and propagation, resulting in filler particle detachment, hence increasing the surface roughness [1]. This outcome is in line with the study conducted by Oliveira et al. and Monteiro et al. [1,30]. It is pertinent to mention that Tetric N Ceram has greater filler particle size (3 micron) as compared to Z350 (1.4 micron) composite filler. This can be explained by the composition of resin composite matrix, which may be less resistant to the dentifrice and tooth-brushing effect [31]. Moreover, it was also reported that whitening toothpastes, irrespective of their chemical composition, causes surface roughness on resin composite surface [32]. Similarly, the effect of tooth-brushing itself on surface roughness cannot be ignored leading to irregularities on the material’s surface [33,34].

In summary, the outcomes of the present study displayed variable influence of dentifrice and toothbrush abrasion exposure on surface roughness, reduced microhardness, and decreased surface gloss of the nano filled composites. Overall, the material type was more influential on the surface properties compared to the type of dentifrices, both showed similar outcomes. It is, therefore, recommended that the type of polymeric composite material is critical for their long-term surface properties, and oral factors, including diet, use of dentifrices, parafunction, and oral hygiene care, will influence properties of nano filled polymers. Oral hygiene reinforcement should be performed in order to maintain aesthetic restoration and durability of nano filled polymeric composites. Further studies assessing their influence on future researches are still required to validate the outcomes of the present study.

## 5. Conclusions

Conventional resin composite (Z350) showed comparable surface hardness and gloss before and after dentifrice exposure, however, micro-roughness increased significantly due to dentifrice exposure. Bulk-fill resin (Tetric N Ceram) showed significant loss of micro-hardness, gloss, and increase in micro-roughness on dentifrice exposure. Conventional nano-hybrid composite polymer showed better durability in resisting loss of surface properties compared to bulk-fill resin polymer in the present experiment.

## Figures and Tables

**Table 1:** Composition of dentifrice and composite materials.

Material	Composition
Colgate Optic White	Propylene Glycol, Calcium Pyrophosphate, PVP, PEG/PPG-116/66 Copolymer, PEG-12, Glycerin, flavor, Hydrogen Peroxide, Sodium Lauryl Sulfate, Silica, Tetrasodium Pyrophosphate, Sodium Saccharin, Disodium Pyrophosphate, Sucralose, Phosphoric Acid, BHT, and water. Sodium Monofluorophosphate 0.76% (0.12% *w/v* fluoride ion) RDA = 100
Colgate Optic White Charcoal	Sodium Fluoride—0.24% (0.15% *w/v* fluoride ion). Water, Hydrated Silica, Sorbitol, Glycerin, PEG-12, Pentasodium Triphosphate, Tetrapotassium Pyrophosphate, Sodium Lauryl Sulfate, flavor, cellulose gum, Cocamidopropyl Betaine, Sodium Saccharin, xanthan gum, charcoal powder, Sodium Hydroxide, Blue 1, Red 40, and Titanium Dioxide. RDA = 135–145
Z350	Bis-GMA, BIS-EMA, UDMA, TEGDMA, and Zirconia/silica (particle size = 20–75 nm, cluster size = 0.6–1.4 mm, 59.5 vol %
Tetric N Ceram	BisGMA, bIsEMA, UDMA, and procrylat. Fillers: Barium glass, Ba-Al-Flourosilicate, mixed oxide, highly dispersed silica, and Ytterbium Triflouride.

**Table 2:** Comparison of Microhardness (VHN) means among study groups.

Study Group	Material Property	Mean ± SD	Mean Difference	*p*-Value
Group 1 (ZW)	Microhardness before	75.54 ± 7.46	5.35	0.14 *
	Microhardness after	70.19 ± 2.63		
Group 2 (ZC)	Microhardness before	73.03 ± 5.01	3.45	0.82 *
	Microhardness after	69.5 ± 6.13		
Group 3 (TW)	Microhardness before	59.19 ± 5.58	13.15	0.000 *
	Microhardness after	46.03 ± 3.16		
Group 4 (TC)	Microhardness before	58.33 ± 5.28	12.31	0.000 *
	Microhardness after	46.02 ± 3.15		

* paired *t*-test, ZC: Z350–Colgate Optic Charcoal, TW: Tetric N Ceram bulk-fill–Colgate Optic White, TC: Tetric N Ceram bulk-fill–Colgate Optic Charcoal, ZW: Z350–Colgate Optic White.

**Table 3:** Comparison of post treatment micro-hardness (VHN) among study groups.

Study Groups	Material Property (Post Treatment)	Mean	*p*-Value *	Mean Difference (Loss of Microhardness)	*p*-Value *
Group 1 (ZW)	Microhardness	70.19	0.061 *	5.35	<0.01 ^β^
Group 2 (ZC)	Microhardness	69.50		3.45	
Group 3 (TW)	Microhardness	46.03		13.15	
Group 4 (TC)	Microhardness	46.02		12.31	

* one-way ANOVA, ZC: Z350–Colgate Optic Charcoal, TW: Tetric N Ceram bulk-fill–Colgate Optic White, TC: Tetric N Ceram bulk-fill–Colgate Optic Charcoal, ZW: Z350–Colgate Optic White, and β: statistical significance.

**Table 4:** Individual group (Inter-group) comparison of post treatment micro-hardness among study groups.

Study Group	Study Group Compared to	*p*-Value §
Group 1 (ZW)	Group 2 (ZC)	0.88
	Group 3 (TW)	<0.01 *
	Group 4 (TC)	<0.01 *
Group 2 (ZC)	Group 3 (TW)	<0.01 *
	Group 4 (TC)	<0.01 *
Group 3 (TW)	Group 4 (TC)	0.99

*t*-test, ZC: Z350–Colgate Optic Charcoal, TW: Tetric N Ceram bulk-fill–Colgate Optic White, TC: Tetric N Ceram bulk-fill–Colgate Optic Charcoal, ZW: Z350–Colgate Optic White, * Statistically significant comparisons, and § Tukey post hoc test.

**Table 5:** Overall influence of material and dentifrice on the micro-hardness (VHN) using two-way ANOVA.

Variable	Sum of Squares	df	Mean Squares	F	Sig.
Material	555.02	1	555.028	27.96	0.000
Dentifrice	14.94	1	14.94	0.75	0.393
Material/dentifrice	2.24	1	2.24	0.11	0.739
Error	555.69	28	19.84		
Total	3477.63	32	_		
Corrected total	1127.91	31	_		

**Table 6:** Comparison of Mean Surface Gloss among study groups.

Study Groups	Material Property	Mean	SD	Mean Difference	*p*-Value §
Group 1 (ZW)	Gloss before	159.06	7.37	4.75	0.34
	Gloss after	154.31	9.66		
Group 2 (ZC)	Gloss before	163.06	6.85	8.13	0.09
	Gloss after	154.93	4.89		
Group 3 (TW)	Gloss before	160.85	7.16	17.7	<0.01 *
	Gloss after	143.15	8.77		
Group 4 (TC)	Gloss before	163.58	7.27	12.89	<0.01 *
	Gloss after	150.69	9.09		

§: Paired *t* test; * denotes statistical significance—ZC: Z350, Colgate Optic Charcoal, TW: Tetric N Ceram bulk-fill, Colgate Optic White, TC: Tetric N Ceram bulk-fill, Colgate Optic Charcoal, and ZW: Z350, Colgate Optic White.

**Table 7:** Comparison of post-treatment surface gloss among study groups.

Study Groups	Material Property (Post-Treatment)	Mean	*p*-Value ^$^
Group 1 (ZW)	Gloss	154.31	0.041 *
Group 2 (ZC)	Gloss	154.93	
Group 3 (TW)	Gloss	143.15	
Group 4 (TC)	Gloss	153.69	

* denotes statistical significance, ^$^ one-way ANOVA—ZC: Z350, Colgate Optic Charcoal, TW: Tetric N Ceram bulk-fill, Colgate Optic White, TC: Tetric N Ceram bulk-fill, Colgate Optic Charcoal, and ZW: Z350, Colgate Optic White.

**Table 8:** Individual group (inter-group) comparison of post-treatment surface gloss among study groups.

Comparison of (Study Group)	Comparison with (Study Group)	*p*-Value ^$^
Group 1 (ZW)	Group 2 (ZC)	0.83
	Group 3 (TW)	<0.01 *
	Group 4 (TC)	0.62
Group 2 (ZC)	Group 3 (TW)	<0.01 *
	Group 4 (TC)	0.72
Group 3 (TW)	Group 4 (TC)	<0.01 *

ZC: Z350, Colgate Optic Charcoal, TW: Tetric N Ceram bulk-fill, Colgate Optic White, TC: Tetric N Ceram bulk-fill, Colgate Optic Charcoal, and ZW: Z350, Colgate Optic White. * Statistically significant comparisons, and ^$^ Repeated measure—post hoc test.

**Table 9:** Influence of material and dentifrice on gloss using two-way ANOVA.

Variable	Sum of Squares	df	Mean Squares	F	Sig.
Material	604.04	1	604.04	7.90	0.000
Dentifrice	78.90	1	78.90	1.03	0.318
Material/dentifrice	143.30	1	143.30	1.90	0.179
Error	2140.19	28	76.43		
Total	---------	32	_		
Corrected total	2968.44	31	_		

**Table 10:** Comparison of mean surface roughness (Ra) before and after treatments among study groups.

Groups	Surface Roughness (Ra)	Mean	SD	Mean Difference	*p*-Value §
Group 1 (ZW)	Ra before	0.69	0.41	0.16	<0.01 *
	Ra after	0.85	0.38		
Group 2 (ZC)	Ra before	0.68	0.29	0.33	<0.01 *
	Ra after	1.01	0.33		
Group 3 (TW)	Ra before	0.57	0.12	0.32	<0.01 *
	Ra after	0.89	0.25		
Group 4 (TC)	Ra before	0.73	0.27	0.21	<0.01 *
	Ra after	0.94	0.39		

§: Paired *t* test; * denotes statistical significance, ZC: Z350, Colgate Optic Charcoal, TW: Tetric N Ceram bulk-fill, Colgate Optic White, TC: Tetric N Ceram bulk-fill, Colgate Optic Charcoal, and ZW: Z350, Colgate Optic White.

**Table 11:** Comparison of post-treatment surface roughness (Ra) among study groups.

Study Groups	Material Property (Post Treatment)	Mean	*p*-Value	Mean Difference	*p*-Value
Group 1 (ZW)	Ra	0.85	<0.01 *	0.16	<0.01 *
Group 2 (ZC)	Ra	1.01		0.33	
Group 3 (TW)	Ra	0.89		0.32	
Group 4 (TC)	Ra	0.94		0.21	

* denotes statistical significance using one-way ANOVA, Surface roughness: Ra-ZC: Z350, Colgate Optic Charcoal, TW: Tetric N Ceram bulk-fill, Colgate Optic White, TC: Tetric N Ceram bulk-fill, Colgate Optic Charcoal, and ZW: Z350, Colgate Optic White.

**Table 12:** Individual group (inter-group) comparison of post-treatment surface roughness (Ra) among study groups.

Comparison of	Comparison with	*p*-Value ^$^
Group 1 (ZW)	Group 2 (ZC)	<0.01 *
	Group 3 (TW)	0.07
	Group 4 (TC)	<0.01 *
Group 2 (ZC)	Group 3 (TW)	<0.01 *
	Group 4 (TC)	0.08
Group 3 (TW)	Group 4 (TC)	0.10

ZC: Z350, Colgate Optic Charcoal, TW: Tetric N Ceram bulk-fill, Colgate Optic White, TC: Tetric N Ceram bulk-fill, Colgate Optic Charcoal, and ZW: Z350, Colgate Optic White. * Statistically significant comparisons, and ^$^ Tukey post hoc test.

**Table 13:** Influence of material and dentifrice on surface roughness using two-way ANOVA.

Variable	Sum of Squares	df	Mean Squares	F	Sig.
Material	0.004	1	0.004	0.04	0.82
Dentifrice	0.007	1	0.007	0.07	0.78
Material/dentifrice	0.14	1	0.141	1.54	0.22
Error	2.56	28	0.092		
Total	4.84	32	_		
Corrected total	2.72	31	_		

## Data Availability

The data are available on contact from the corresponding author.
